# May Sulfonamide Inhibitors of Carbonic Anhydrases from *Mammaliicoccus sciuri* Prevent Antimicrobial Resistance Due to Gene Transfer to Other Harmful Staphylococci?

**DOI:** 10.3390/ijms232213827

**Published:** 2022-11-10

**Authors:** Viviana De Luca, Simone Giovannuzzi, Claudiu T. Supuran, Clemente Capasso

**Affiliations:** 1 Institute of Biosciences and Bioresources, CNR, Via Pietro Castellino 111, 80131 Napoli, Italy; 2 Section of Pharmaceutical and Nutraceutical Sciences, Department of Neurofarba, University of Florence, Via U. Schiff 6, Sesto Fiorentino, 50019 Florence, Italy

**Keywords:** carbonic anhydrase, sulfonamides, inhibitors, antimicrobial resistance, antibacterials

## Abstract

*Mammaliicoccus sciuri*, previously known as *Staphylococcus sciuri*, is a Gram-positive bacterium involved in gene transfer phenomena that confer resistance to multiple antibiotics. These plasmid-encoded genes can be easily transferred to other pathogenic staphylococci. Because antibiotic resistance is rising, inhibiting *M. sciuri* proliferation may be a credible strategy for restricting antimicrobial resistance gene transfer to other pathogenic bacteria. Recently, it has been shown that blocking bacterial carbonic anhydrases (CAs, EC 4.2.1.1), metalloenzymes sustaining bacterial metabolic activities, can reduce pathogen survival and fitness. Here, the recombinant *M. sciuri* γ-CA (MscCAγ) has been cloned and purified, utilizing the DNA recombinant technology. Its kinetic properties for the CO_2_ hydration reaction, as well as the sulfonamide inhibition profile, were investigated and compared with those reported earlier for MscCAβ (previously described as SauBCA) and the two off-target human CA isoforms (hCA I and hCA II). The recombinant MscCAγ showed significant hydratase activity. Moreover, the MscCAγ sulfonamide inhibitory profile was different from that of MscCAβ, implying that a varied amino acid set typifies the catalytic pocket of the two enzymes. These differences provide additional evidence for the possibility of developing novel CA class-specific inhibitors.

## 1. Introduction

Humans and animals are reservoirs for several species of staphylococci [1], such as *Staphylococcus aureus*, which is found in 15–30% of healthy individuals in the nasal passages and throat, as well as smaller amounts in the digestive tract and perineum [2,3]; *S. saprophyticus* colonizes the urinary tract [4]; *S. capitis* is present on the scalp [5]; *S. epidermidis* is a skin commensal in nearly 100% of humans [6]; *S. auricularis* is found around and in the external ear canal [7]; whereas bovine mammary secretions also contain Staphylococci [8]. *S. aureus* is the most pathogenic species among the staphylococci. It is responsible for various infections, such as pneumonia, deep abscesses, osteomyelitis, endocarditis, phlebitis, mastitis, and meningitis, and is often a significant cause of nosocomial infections [9,10,11,12,13]. The bacteria *S. aureus* and *S. epidermidis* are becoming increasingly frequent in cases of multidrug resistance, mainly because methicillin-resistant *S. aureus* creates outbreaks in hospitals and can potentially become epidemic. In 2020, combining phylogenetic data, Madhaiyan et al. reclassified some members of the staphylococci group (*S. sciuri*, *S. fleurettii*, *S. lentus*, *S. stepanovicii*, and *S. vitulinus*) into the new genus Mammaliicoccus [14], having *Mammaliicoccus sciuri* as the type species [14]. This species is frequently found in the milk samples collected from dairy farms and calves’ nasal swabs. *M. sciuri* is highly important because it harbors antimicrobial resistance genes against several antibiotics [15,16,17]. These genes are found on mobile genetic components such as plasmids, making them easily transmissible to other bacteria, including staphylococci [17]. Infections in humans and animals that are resistant to treatment may be increased if resistance genes are passed on from *mammaliicocci* to other species, especially more dangerous members of the Staphylococcaceae family, such as *S. aureus*. Based on these concerns, and because antibiotic resistance is currently on the rise, preventing the proliferation of *M. sciuri* may be considered a valid alternative for blocking the antimicrobial resistance gene transfer from this pathogen to other pathogenic bacteria.

Recently, several papers have demonstrated that pathogen survival/fitness may be impaired by inhibiting bacterial carbonic anhydrases (CAs, EC 4.2.1.1) [18,19,20,21,22,23,24,25,26,27]. CAs are ubiquitous metalloenzymes that catalyze the reversible CO_2_ hydration reaction, balancing the endogenous levels of carbon dioxide (CO_2_), bicarbonate (HCO_3_^−^), and protons (H^+^), which are essential for sustaining bacterial metabolic activities. This is the main reason to consider CAs as attractive druggable targets, and fortunately, many specific chemical classes of CA inhibitors (CAIs) exist, some of which are FDA-approved drugs [28,29,30,31]. The eight CA classes across all life kingdoms are denoted by the Greek letters α, β, γ, δ, ζ, η, θ, and ι [32,33,34,35,36]. Currently, four CA-classes (α, β, γ, and ι) have been found in bacteria [37].

In 2016, the *M. sciuri* β-CA was cloned, expressed, and purified and was erroneously attributed to *S. aureus* [38,39] due to errors in the sequence annotations found in databases. The biocatalyst was indicated with the SauBCA (from *S. aureus* β-CA) acronym, showed excellent CO_2_ hydrase catalytic activity, and was amenable to inhibition by sulfonamides and anions [38,39]. Exploring the *M. sciuri* genome, a CA-gene encoding for a γ-class CA has also been identified. Here, the recombinant *M. sciuri* γ-CA (MscCAγ) has been produced utilizing the DNA recombinant technology, and its kinetic properties were examined using a stopped-flow CO_2_ hydrase technique. In addition, a comprehensive study was conducted on the inhibition profiles of MscCAγ utilizing the substituted benzene-sulfonamides and clinically licensed sulfonamide/sulfamate/sulfamide drugs that belong to the classical CAIs. Generally, CAIs inhibit mammalian α-CAs in the nanomolar range and have been used clinically for decades as antiglaucoma, diuretic, antiepileptic, antiobesity, and anticancer agents [28,29,30,31].

The impairment of the *M. sciuri* growth through the inhibition of its carbonic anhydrases (β and γ-CAs) may help in countering bacterial antibiotic resistance because the CAIs act across a mechanism different from that of the common antibiotics and, at the same time, may represent an approach to avoid the transmission of antibiotic resistance genes to other harmful bacteria, such as those escaping the commonly used antibiotics.

## 2. Results and Discussion

### 2.1. Gene Identification, Sequence Alignment, and Phylogenetic Analysis of γ-CA Encoded by the M. sciuri Genome

The gene responsible for the hydratase activity of the *γ*-CA was identified by exploring the genome of *M. sciuri* deposited in the NCBI datasets (https://www.ncbi.nlm.nih.gov/genome/?term=sciuri (accessed on 9 July 2022)). It encodes a protein (MscCAγ) of 169 aa (Sequence ID: WP_049318612.1), displaying high amino acid sequence similarities to γ-CAs identified in the genome of other Mammaliicoccus species, such as the CA from *M. vitulinus* (Sequence ID: WP_103322725.1, 85% of identity, 168 aa), *M. stepanovicii* (Sequence ID: WP_095087344.1, 81% of identity, 170 aa) and *M. lentus* (Sequence ID: WP_257504613.1, 81% of identity, 169 aa). MscCAγ reduced its sequence similarity compared to γ-CAs amino acid sequences identified in species different from Mammaliicoccus. For example, MscCAγ shows an identity of 46% for the γ-CA (EcoCAγ) from *Escherichia coli* (Sequence ID: MSL98108.1, 173 aa), 38% for the γ-CA (PgiCAγ) from *Porphyromonas gingivalis* (Sequence ID: WP_004584482.1, 179 aa) and 40% in the case of the *Vibrio cholerae* γ-CA (VchCAγ) (Sequence ID: WP_000095101.1, 184 aa).

**Figure 1:**
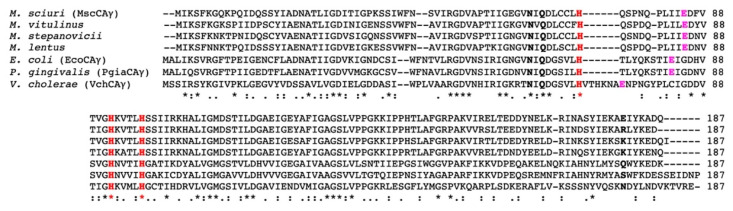
An alignment of the amino acid sequences of the γ-CAs from different bacterial sources, such as *M*. *sciuri*, *M. vitulinus*, *M. stepanovicii*, *M. lentus*, *E. coli*, *P. gingivalis*, and *V. cholerae*. The γ-CA hallmarks, such as the metal ion ligands, the residues which participate in a network of hydrogen bonds, and the proton shuttle residue, are shown in red bold, black bold, and violet bold, respectively. The asterisk (*) indicates identity at all aligned, the symbol (:) relates to conserved substitutions, while (.) means semi-conserved substitutions.

The analysis of these CAs by multiple sequence alignment revealed that all the amino acid hallmarks, which typify the γ-CAs, are conserved in the MscCAγ enzyme, such as the three histidine residues responsible for the binding of the metal ion, the residues which participate in a network of hydrogen bonds with the catalytic water molecule, and the proton shuttle residue, which is a glutamic acid residue and not a histidine as in the α-CAs (Figure 1).

**Figure 2:**
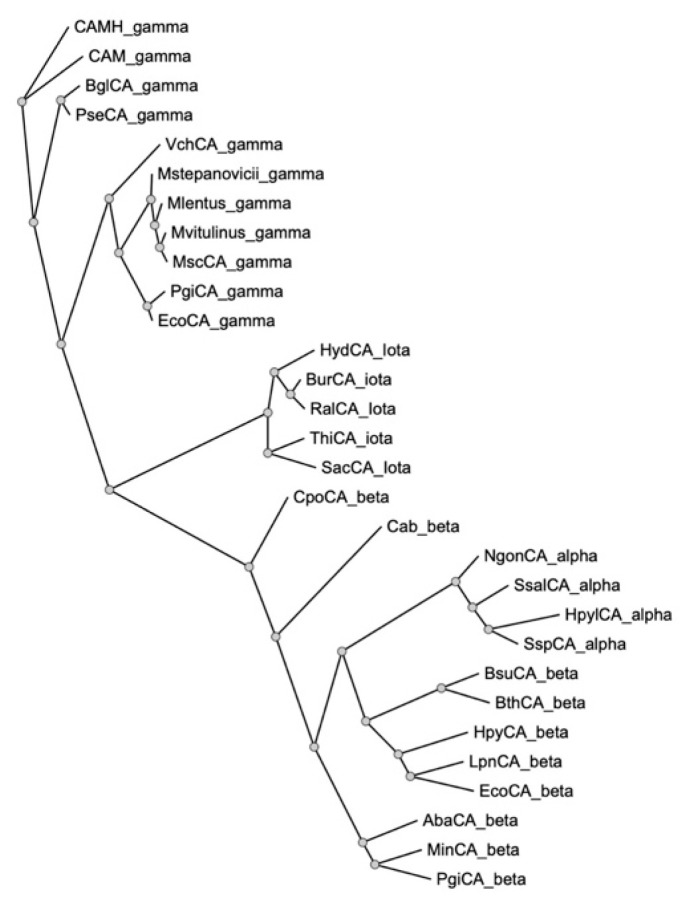
The phylogenetic tree was constructed using the software PhyML 3.0 and using α-, β-, γ-, and 𝜄-CAs identified in different bacterial species. Legend: CAMH_gamma, *Methanosarcina thermophila* TM-1, ACQ57353.1; CAM_gamma, *Methanosarcina thermophila*, WP_052721819.1; BglCA_gamma, *Burkholderia gladioli*, WP_013697304.1; PseCA_gamma, *Pseudomonas* sp., WP_010168409.1; VchCA_gamma, *Vibrio cholerae*, WP_000095101.1; Mstepanovicii_gamma, *Mammaliicoccus stepanovicii*, WP_095087344.1; Mlentus_gamma, *Mammaliicoccus lentus*, WP_257504613.1; Mvitulinus_gamma, *Mammaliicoccus vitulinus*, WP_103322725.1; MscCA_gamma, *Mammaliicoccus sciuri*, WP_049318612.1; PgiCA_gamma, *Porphyromonas gingivalis*, WP_004584482.1; EcoCA_gamma, *Escherichia coli*, MSL98108.1; HydCA_Iota *Ephemeroptericola cinctiostellae*, WP_114562659.1; BurCA_iota, *Burkholderia territorii*, WP_063553346.1; RalCA_Iota, *Ralstonia* sp., WP_089190700.1; ThiCA_iota, *Thiotrichales bacterium*, OYX05505.1; SacCA_Iota *Saccharothrix* sp., WP_053720260.1; CpoCA_beta, *Candidatus Prometheoarchaeum syntrophicum*, WP_147661847.1; Cab_beta, *Methanothermobacter thermautotrophicus*, 1G5C_A; NgonCA_alpha, *Neisseria gonorrhoeae*, WP_003688976.1; SsalCA_alpha, *Streptococcus salivarius*, WP_002888224.1; HpylCA_alpha, *Helicobacter pylori*, WP_010882609.1; SspCA_alpha, *Sulfurihydrogenibium* sp., HBT99398.1; BsuCA_beta, *Brucella suis*, AAN33967.1; BthCA_beta, *Burkholderia thailandensis*, WP_009893276.1; HpyCA_beta, *Helicobacter pylori*, BAF34127.1; LpnCA_beta, *Legionella pneumophila*, WP_011946835.1; EcoCA_beta, *Escherichia Coli*, WP_047081292.1; AbaCA_beta, *Acinetobacter baumannii*, WP_001141692.1; MinCA_beta, *Myroides injenensis*, WP_010254382.1; PgiCA_beta, *Porphyromonas gingivalis*, WP_012458351.1.

A phylogenetic tree was created by the maximum-likelihood phylogeny software PhyML [40] using the deduced amino acid homolog sequences of the bacterial α-, β-, γ-, and 𝜄-CAs found in the NCBI datasets. As expected from this analysis, the four CA classes that have been found in bacteria clustered separately since they are phylogenetically distinct families, with the γ-class resulting in the ancestral class (Figure 2) [32,35,41], and the α and β CAs, the most recent families. The *Mammaliicoccus* γ-CAs were closely related to one another as well as the other bacterial CAs, such as those identified in *E. coli*, *P. gingivalis*, and *V. cholerae* (Figure 2).

### 2.2. The Expression of the MscCAγ and Its Subsequent Purification

**Figure 3:**
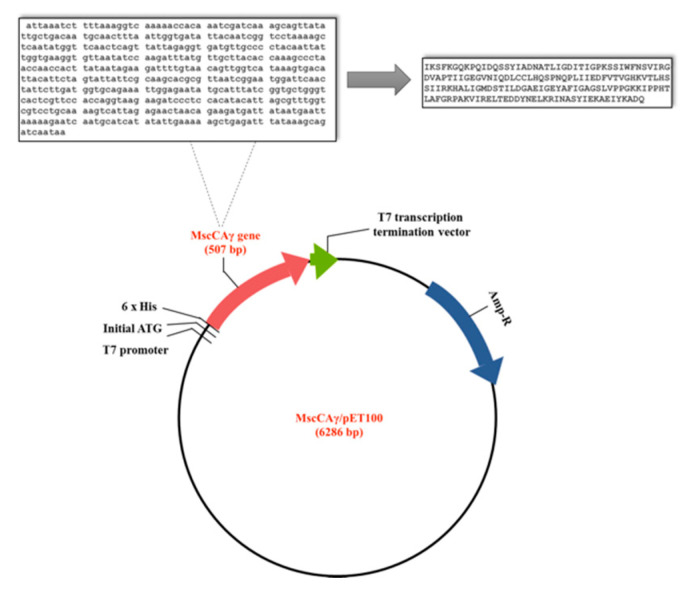
Schematic representation of the obtained construct for overexpressing the recombinant MscCAγ. The cloned nucleotide sequence (**left**) and the encoded amino acid sequence (**right**) are reported at the top of the figure. The bottom of the figure shows the construct with the T7 promoter to control the expression of the heterologous gene in *E. coli*, the initiation ATG in the N-terminal tag, the 6xHis tag, allowing the purification of the fusion protein with a metal-chelating resin, the gene of 507 bp, encoding for the MscCAγ, and the T7 transcription terminator, which permits an efficient transcription termination.

The gene of 507 bp, encoding for the MscCAγ, including the four base pair sequences CACC at the 5′ end, necessary for its directional cloning, and with no modification at the 3′ end, was cloned in the expression vector pET100 D-TOPO (Figure 3).

**Figure 4:**
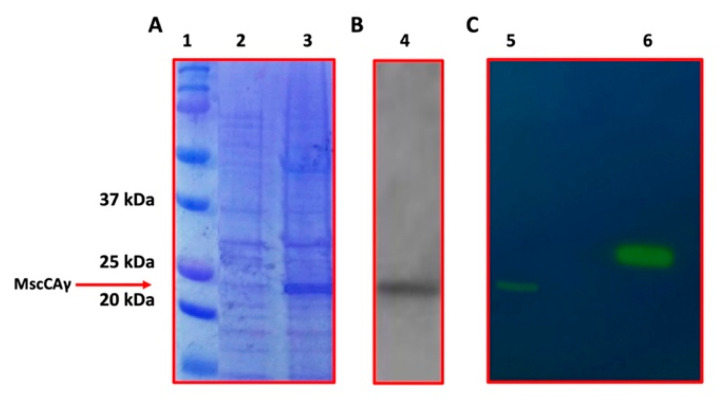
SDS-PAGE electropherogram (**A**), Western-Blot (**B**), and Protonogram (**C**). Legend: Lane 1, molecular markers, molecular mass values starting from the top: 150, 100, 75, 50, 37, 25, 20, and 15 kDa; Lane 2: cellular extract before induction with IPTG; Lane 3, MscCAγ overexpression induced by IPTG addition; Lane 4, MscCAγ band after affinity chromatography purification and detected by His-tag antibody; Lane 5, MscCAγ hydratase activity, which is denoted by the yellow band due to the CO_2_ hydration reaction catalyzed by the enzyme on the polyacrylamide gel and responsible of the pH variation from 8.2 to the transition point of the dye; Lane 6, commercial bovine CA, used as positive controls.

The obtained recombinant plasmid, MscCAγ/pET100, was transformed into *E. coli* BL21(DE3) cells. Ampicillin transformant colonies containing MscCAγ/pET100 were screened by colony PCR to confirm the presence of the gene encoding the MscCAγ enzyme. The MscCAγ/pET100 transformants were grown in an LB-ampicillin medium. MscCAγ overexpression was induced by adding 0.5 mM IPTG and culturing the cell at 20 °C for 18 h. The obtained cell extract containing the produced recombinant protein with six his-tags at the C-terminus was purified by affinity chromatography using a column packed with a nickel affinity gel to selectively isolate a tag of six or more histidine residues (His-Tag). The purified enzyme was analyzed by SDS-PAGE and Western-Blot, to verify the overexpression of the soluble target protein, whose activity was checked qualitatively using the protonography technique [42,43,44] (Figure 4).

As presented in Figure 4A,B, the SDS-PAGE and WB evidenced one band corresponding to a monomer with an apparent molecular mass of about 22.0 kDa. The His-tagged recombinant enzyme was predicted to have a molecular mass of 23.2 kDa. The MscCAγ protonography analysis allowed the qualitative determination of the enzyme hydratase activity on a polyacrylamide gel. The pH change caused by the CO_2_ hydration reaction has been shown as a yellow band in the protonogram (Figure 4C), indicating that MscCAγ is an active SDS-PAGE migrating biocatalyst with a molecular mass of 22.0 kDa (Figure 4C). This method produced about 3.5 mg of enzyme per 3 g wet biomass with a purity higher than 95%.

### 2.3. Kinetic Parameters Determined Using the Stopped-Flow Method

With CO_2_ as a substrate and stopped-flow spectrophotometry (a method for analyzing rapid reactions in solution), the kinetic parameters for MscCAγ were calculated for the physiologic reaction catalyzed by this enzyme (Table 1).

The reported mean values are from three independent assays performed by the stopped-flow technique; errors ranged from ±5–10% of the reported values.

Table 1 shows a comparison of the kinetic parameters of recombinant MscCAγ with those obtained for the β-CA (MscCAβ (ex SauBCA)) from *M. sciuri* and the two human α-CA isoenzymes (hCA I and hCA II). MscCAγ had a significant catalytic activity with a k_cat_ value of 6.2 × 10^5^ s^−1^, resulting in 4.13 times and 3.10 faster than the β-CA (k_cat_ = 1.5 × 10^5^ s^−1^), previously identified in the *M. sciuri* by Urbanski et al. [38], and the human isoform hCA I (k_cat_ = 2.0 × 10^5^ s^−1^), respectively. On the contrary, MscCAγ was about 2.25 times slower than the human isoform hCA II (k_cat_ = 1.4 × 10^6^ s^−1^), considered one of the faster known CAs [45]. Not surprisingly, MscCAγ and MscCAβ exhibited a difference in their inhibition behavior for the classical primary sulfonamide inhibitor acetazolamide (AAZ) (Table 1) since, generally, enzymes belonging to different CA families do show this behavior [32,33,34,35]. AAZ was a better inhibitor of MscCAγ with an inhibition constant (K_I_) value of 245 nM with respect to the value obtained for MscCAβ (K_I_ = 628 nM). Instead, the hCA II activity was highly inhibited by AAZ, with an inhibition constant of 12 nM. Because mammals contain only α-CAs, these discoveries might open the path for designing highly selective drugs that do not interfere with human CAs.

**Figure 5:**
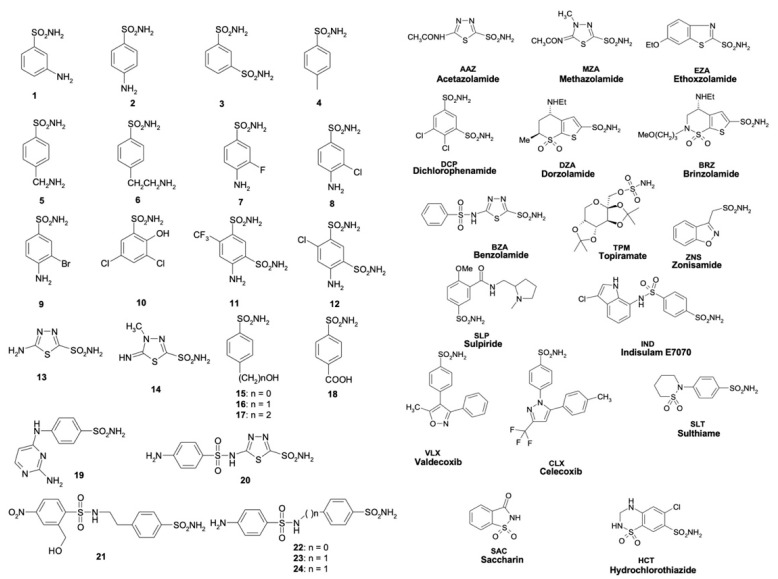
The structures of sulfonamide, sulfamate and *sulfamate* CAIs (**1**–**24** and **AAZ-HCT**) were investigated to inhibit bacterial and human CAs.

In this context, using a stopped-flow technique and as reported in the next paragraph, the inhibition profile of MscCAγ has been explored with a variety of substituted benzene-sulfonamides and therapeutically licensed drugs belonging to the classes of conventional CAIs (Figure 5).

### 2.4. Sulfonamide Inhibition Profile

Sulfonamides are the most significant CAIs among the other chemical families reported in the scientific literature [46]. They are very efficient as antibacterial, as demonstrated by the fact that several FDA-approved CAIs have been modified to target vancomycin-resistant enterococci (VRE) [21,47,48], kill *Helicobacter pylori* in vitro [20], and inhibit *Neisseria gonorrhoeae* in vitro and in vivo in mouse models of gonococcal genital tract infection [18]. Recently, we have used *E. coli* cells as a model system to examine the impact of carbonic anhydrase inhibition on bacterial growth [37]. It has been found that AZA, a sulfonamide FDA-approved CA inhibitor, impairs bacterial growth as well as the uptake of glucose, which *E. coli* (and other bacteria) use as a carbon source for growth and metabolism [37]. Such knowledge is highly sought after since it could lead to the development of new pharmacological medicines capable of inhibiting the growth and virulence of bacteria with a novel mode of action (inhibition of the CAs), free of the drug resistance that plagues the multiple classes of medicines used in clinical practice. The inhibition of the CA enzymes from *M. sciuri* could thus compromise the growth of the bacterium and, thus, the transmission of antibiotic-resistance genes to other pathogenic bacteria.

Table 2 compares the sulfonamide inhibition profiles of MscCAγ, MscCAβ (ex SauBCA), and the two human isoforms (hCA I and hCA II).

Only six sulfonamides utilized to evaluate the MscCAγ inhibition profile had a K_I_ less than 100 nM. Compounds **21**, **22**, **24**, **MZA**, **EZA**, and **DZA** are examples of this. On the contrary, the *M. sciuri* CA belonging to the β-class (MscCAβ) was inhibited below 100 nM by the following twelve inhibitors: **3**, **4**, **7**, **8**, **9**, **11**, **12**, **20**, **21**, **22**, **23**, and **24**. Interestingly, **MZA**, **EZA**, and **DZA** resulted in effective inhibitors of MscCAγ (K_Is_ in the range of 24.5–96.6 nM) and moderate inhibitors of MscCAβ with (K_Is_ = 698–909 nM). The sulfonamide inhibitory profile of the two human α-CAs shows distinct behavior, too. In fact, the human isoform hCA II was more susceptible to these inhibitors than the isoform hCA I (Table 2). These findings corroborate the distinct spatial arrangement of the catalytic pocket among CA classes and imply a high likelihood of success in developing novel CA class-specific inhibitors. The structural mechanisms causing the K_I_ fluctuations can only be explained by studying the three-dimensional structures of MscCAγ and MscCAβ, which are currently unavailable.

Most of the tested inhibitors had K_I_ values between 176 and 923 nM for MscCAγ and 193 and 909 nM for MscCAβ, as shown in Table 2, resulting in moderate inhibitors of the two bacterial enzymes. Inhibitors with similar K_I_ values for both bacterial CAs include **1**, **2**, **5**, **6**, **13**, **14**, **16**, **17**, **18**, **BZA**, **TPM**, **IND**, and **SLT**. Some of these inhibitors showed high sensitivity against the hCA II human isoform but had little effect against hCA I (K_IS_ > 10,000 nM). The two FDA-approved drugs, **BZA** and **ZNS,** were only moderately efficient in inhibiting the bacterial enzymes (K_IS_ in the range 444–455 nM), but they had a significant inhibitory impact on the human isoenzymes (K_IS_ in the range 9.0–56.0 nM).

Some inhibitors were not particularly powerful or effective against MscCAγ with K_Is_ in the range of 32,978–1540 nM. They are the substituted benzene-sulfonamides, such as **7**, **10**, **11**, **12**, **15**, **19**, **20**, **DCP**, **SLP**, **VLX**, **CLX**, **SAC,** and **HCT.**

The variation in K_I_ values seen in the sulfonamide inhibition profile reported in Table 2 suggests that it may be possible to construct efficient and selective inhibitors of the bacterial enzymes that do not impede the activity of the human CAs, despite the great degree of similarity of the amino acid residues contouring the catalytic pocket.

## 3. Materials and Methods

### 3.1. Bacterial Species, Vectors, and Chemicals

Initial cloning was carried out in *E. coli* DH5α cells (Agilent, Santa Clara, CA, USA), while heterologous expression of the recombinant *M. sciuri* γ-CA (MscCAγ) was carried out in *E. coli* BL21 (DE3) cells (Agilent, Santa Clara, CA, USA). Specifically, we used the pET100/D-TOPO vector from Invitrogen (Carlsbad, CA, USA) to express the recombinant protein as a fusion protein with a 6-histidine tag at the N-terminus. Merck was the source of the Luria Bertani Broth (LB), ampicillin, and other chemicals (Darmstadt, Germany).

### 3.2. Screening of Protein Databases, Sequence Analysis, and Phylogenetic Analysis

NCBI-BLASTP, a sequence analysis tool developed for searching protein databases, has been used to identify *M. sciuri* γ-CA [49,50]. A γ-CA sequence previously identified in a bacterial specie was utilized as a query sequence to search for proteins. The NCBI-BLASTP program found a highly similar amino acid sequence to the homologous enzyme, which was given the accession number WP_049318612.1 (NCBI Reference Sequence) in the created output file, which corresponded to the *M. sciuri* γ-CA. The alignment of the main structure of the proteins considered in the present manuscript was performed using the program CLUSTAL OMEGA, which was developed for doing the multiple alignments of protein sequences [51]. A phylogenetic dendrogram was generated using the program NGPhylogeny, which sought out the tree with the highest probability [52].

### 3.3. Cloning, Expression of the MscCAγ, and Its Subsequent Purification

The encoding MscCAγ synthetic gene had the NdeI and XhoI restriction sites at the 5′ and 3′ ends, respectively, as well as 4 base-pair sequences (CACC) required for directed cloning at the corresponding 5′ end. The synthetic MscCAγ was ligated into the expression vector pET100/D-TOPO (Invitrogen, Carlsbad, CA, USA) to create the expression vector MscCAγ/pET100, which contains a nucleotide sequence encoding for a polypeptide with an additional 6 histidines before the insertion point to aid in target protein purification. Bidirectional automated sequencing validated the integrity of the MscCAγ gene and the absence of mistakes in the ligation sites. MscCAγ/pET100 was used to transform competent *E. coli* BL21 (DE3) cells (Agilent, Santa Clara, CA, USA). A single colony of transformed *E. coli* BL21 (DE3) was incubated overnight in a 10 mL Luria-Bertani broth (LB) medium containing ampicillin (100 mg/mL) at 37 °C with continual agitation in a shaking incubator (200 rpm). The following day, 5 mL of cultivated materials were extracted and inoculated in 1 L of LB broth. The culture was cultivated at 37 °C with vigorous shaking (200 rpm) at an OD_600nm_ of 0.6. After 30 min of incubation, 0.5 mM isopropyl-β-D-thiogalactopyranoside (IPTG) was added, followed by 0.5 mM ZnSO_4_. The incubation period lasted an additional 20 h at 20 °C and 200 rpm shaking. The bacterial suspension was then tested and analyzed on 12% SDS-PAGE to confirm MscCAγ overexpression. SDS-polyacrylamide gel electrophoresis (SDS-PAGE) with 12% gel was done as described by Laemmli [53]. Twenty hours after induction, cells were collected and broken up using ultrasound at 4 °C. After centrifugation, a 1.0 mL/min flow of 0.02 M phosphate buffer (pH 8.0) containing 0.01 M imidazole and 0.5 M KCl was used to load the supernatant onto HIS-Select HF Nickel Affinity Gel (Sigma-Aldrich, St. Louis, MO, USA). 0.02 M phosphate buffer (pH 8.0) containing 0.5 M KCl and 0.3 M imidazole was used to elute the recombinant MscCAγ protein from the column at a flow rate of 1.0 mL/min. Active fractions were collected, dialyzed, concentrated, and subjected to SDS-PAGE analysis. Protein content was measured using the Bradford method (Bio-Rad) [54]. Western-Blot and protonography were performed as previously described [55].

### 3.4. Enzyme Assay

An applied photophysics stopped-flow instrument has been used for assaying the CA-catalysed CO_2_ hydration activity [56]. Phenol red (at a concentration of 0.2 mM) has been used as an indicator in a buffer containing 20 mM Tris (pH 8.3), 20 mM NaClO_4_ (for maintaining a constant ionic strength), measuring the absorbance maximum of 557 nm, and following the initial rate of the CA-catalysed CO_2_ hydration reactions for a period of 10–100 s. The CO_2_ concentrations values ranged from 1.7 to 17 mM during the determination of the kinetic parameters. For the determination of the inhibition constants, at least six traces of the initial 5–10% of the reaction have been used for determining the initial velocity. The uncatalyzed rates were determined similarly and subtracted from the total observed rates. Stock solutions of inhibitor (10–50 mM) were prepared in distilled-deionized water, and dilutions up to 0.01 mM were done thereafter in the assay buffer. The inhibitor and enzyme solution were preincubated together for 15 min at room temperature to allow the formation of the E-I complex or for the eventual active site-mediated hydrolysis of the inhibitor. As reported earlier, the inhibition constant values were obtained by non-linear least-squares methods using PRISM 3 and represent the mean from at least three different determinations [57].

## 4. Conclusions

The name Mammaliicoccus relates to the ecological niche from which these bacteria are frequently isolated as a common resident of the skin of a diverse range of farm and wild mammals. Recently, great importance has been attributed to *M. sciuri*, primarily because it may function as a source of resistance and a virulence determinant [15,16,17]. It has been proposed that *M. sciuri* is a significant vector of virulence and antibiotic-resistance genes that may be transferred to *S. aureus* and other clinically significant staphylococcal species [15,16,17]. The development of antimicrobial resistance in bacteria is a growing public health concern that puts at elevated risk many advances in contemporary medicine. Thus, it is critical to monitor the resistance situation in human and veterinary pathogens as well as in other bacteria, which potentially may pass genes of antibiotic resistance to other pathogenic bacteria. In this context, suppressing *M. sciuri* growth is a reasonable tactic for halting the spread of antibiotic-resistant gene transfer. Here, is investigated the possibility of inhibiting in vitro the *M. sciuri* CAs, which are critical metalloenzymes in sustaining bacterial metabolic activities. The genome of *M. sciuri* encodes for two CAs, MscCAγ and MscCAβ (ex-SauBCA), which belong to the γ- and β-CA classes, respectively. The two CAs showed significant catalytic activity with a k_cat_ in the order of 10^5^ s^−1^ and were susceptible to inhibition by sulfonamides. In particular, six sulfonamides, compounds **21**, **22**, **24**, **MZA**, **EZA**, and **DZA,** resulted in being effective inhibitors of MscCAγ (K_I_ less than 100 nM), while MscCAβ showed 12 inhibitors (**3**, **4**, **7**, **8**, **9**, **11**, **12**, **20**, **21**, **22**, **23**, and **24**) with this range of K_I_. The main results emerging from the sulfonamide inhibition profile were that the two *M. sciuri* enzymes showed different K_I_ values when compared to each other, as well as with the two human isoforms (hCA I and hCA II), suggesting that distinct amino acids align the catalytic pocket among different CA classes. These corroborate the possibility of developing novel CA class-specific inhibitors with a high likelihood of success. Moreover, by investigating the three-dimensional structures of MscCAβ and MscCAγ, which our laboratories are attempting to resolve through X-ray crystallographic studies, it will be possible to develop novel inhibitors highly selective for the two bacterial enzymes without interfering with the activity of the human CAs.

## Figures and Tables

**Table 1:** MscCAβ and MscCAγ kinetic parameters were compared with those obtained for the two human α-CA isoenzymes (hCA I and hCA II). The β and γ-class enzymes were investigated for the CO_2_ hydration reaction in 20 mM Tris buffer pH 8.3 and 20 mM NaClO_4_ at 25 °C, whereas the α-CAs in 20 mM Hepes buffer, pH 7.4 and 20 mM NaClO_4_ at 25 °C.

Organism	Acronym	Class	k_cat_ (s^−1^)	k_cat_/K_m_ (M^−1^ × s^−1^)	K_I_ (Acetazolamide) (nM)
*Homo sapiens*	hCA I	α	2.0 × 10^5^	5.0 × 10^7^	250
	hCA II	α	1.4 × 10^6^	1.5 × 10^8^	12
*Mammaliicoccus sciuri*	MscCAβ (ex SauBCA)	β	1.5 × 10^5^	2.6 × 10^7^	628
	MscCAγ	γ	6.2 × 10^5^	9.5 × 10^6^	245

**Table 2:** Inhibition profile of the β- and γ-CAs from *M. sciuri* and the two isoforms hCA I and hCA II from *Homo sapiens* with sulfonamides **1**–**24** and the clinically used drugs **AAZ**-**HCT**. A stopped-flow assay was used for the analysis.

Inhibitor	K_I_ (nM) ^a^			
	hCA I	hCA II	MscCAγ	MscCAβ (ex SauBCA)
**1**	28,000	300	797	355
**2**	25,000	240	888	409
**3**	79.0	8.0	552	95
**4**	78,500	320	647	83
**5**	25,000	170	354	193
**6**	21,000	160	176	253
**7**	8300	60.0	2010	93
**8**	9800	110	426	95
**9**	6500	40.0	478	75
**10**	7300	54.0	2429	202
**11**	5800	63.0	32,978	81
**12**	8400	75.0	17,200	79
**13**	8600	60.0	539	417
**14**	9300	19.0	820	553
**15**	5500	80.0	2931	619
**16**	9500	94.0	915	603
**17**	21,000	125	736	232
**18**	164	46.0	722	555
**19**	109	33.0	1893	909
**20**	6.0	2.0	1626	92
**21**	69.0	11.0	83.4	85
**22**	164	46.0	72.5	83
**23**	109	33.0	217	92
**24**	95.0	30.0	45.7	96
**AAZ**	250	12.0	245	628
**MZA**	50.0	14.0	94.5	863
**EZA**	25.0	8.0	96.6	698
**DCP**	1200	38.0	8580	-
**DZA**	50,000	9.0	43.8	909
**BRZ**	45,000	3.0	100	815
**BZA**	15.0	9.0	444	501
**TPM**	250	10.0	578	466
**ZNS**	56.0	35.0	923	4551
**SLP**	1200	40.0	2203	807
**IND**	31.0	15.0	369	588
**VLX**	54,000	43.0	1540	509
**CLX**	50,000	21.0	3024	871
**SLT**	374	9.0	405	824
**SAC**	18,540	5959	29,687	667
**HCT**	328	290	18,346	593

^a^ Mean from three different assays using a stopped-flow technique (errors were in the range of ± 5–10% of the reported values).

## Data Availability

Not applicable.
